# “They choke to death in front of your very eyes”: nurses’ lived experiences and perspectives on end-of-life care during COVID-19

**DOI:** 10.1186/s12904-024-01352-3

**Published:** 2024-02-08

**Authors:** Daniel Sperling

**Affiliations:** Department of Nursing, University of Haifa, Haifa, Israel

**Keywords:** COVID-19, Nurses, End-of-life, Death and dying, Family members, Advance care directives, Caring for the body of a deceased, Life-saving treatment, Moral distress, Israel

## Abstract

**Background:**

The COVID-19 pandemic led to an intensified fear and threat of dying, combined with dying and grieving in isolation, in turn significantly impacting nursing in end-of-life situations. The study aims (1) to understand the lived experiences of nurses who provided care to end-of-life patients during COVID-19; and (2) to explore whether providing care under such circumstances altered the perspectives of these nurses regarding end-of-life care.

**Methods:**

Applying the phenomenological-interpretive qualitative approach, 34 in-depth semi-structured interviews were conducted between March 2020-May 2021 with nurses from eight hospitals in Israel who were recruited through purposive and snowball sampling. Thematic analysis was applied to identify major themes from the interviews.

**Results:**

Five main themes emerged from the analysis, including: (1) a different death; (2) difficulties in caring for the body after death; (3) the need for family at end-of-life; (4) weaker enforcement of advance care directives; and (5) prolonging the dying process.

**Discussion:**

During the pandemic, nurses encountered numerous cases of death and dying, while facing ethical and professional issues regarding end-of-life care. They were required to administer more aggressive care than usual and even necessary, leading to their increased moral distress. The nurses’ ethical concerns were also triggered by the requirement to wrap the corpse in black garbage-like bags to prevent contagion, which they felt was abusing the dead. The findings also demonstrate how family presence at end-of-life is important for the nursing staff as well as the patient. Finally, end-of-life situations during the pandemic in Israel were managed in an individual and personal manner, rather than as a collective mission, as seen in other countries.

**Conclusions:**

The study offers insights into the nurses’ attitudes towards death, dying, and end-of-life care. An emphasis should be placed on the key elements that emerged in this study, to assist nurses in overcoming these difficulties during and after medical crises, to enhance end-of-life care and professionalism and decrease burnout.

**Supplementary Information:**

The online version contains supplementary material available at 10.1186/s12904-024-01352-3.

## Background

The global COVID-19 pandemic has had far reaching implications on the health and wellbeing of vast numbers of people worldwide. It is marked by an environment of high mortality rates, recently termed as ‘deathscape’ [1]. Such a work environment has led to intense experiences relating to fear of dying, threat of dying, and both dying and grieving in isolation – without the care and comfort of loved ones [2]. The social distancing and isolation protocols applied in hospitals and care homes often rendered patients dying alone, in hospitals, hospices, and even at home [3]. Yet not only were people stripped of their right to not die alone, but they were also subjected to stigmatization in the public discourse and the media, as dying alone is considered a non-normative event [4].

The pandemic also led to restrictions on transferring patients between facilities, and to insufficient time and opportunities for discussing advance care planning [5], including uncertain clinical outcomes, rapid patient deterioration, infection control protocols, etc. [6]. Overall, the pandemic significantly worsened the capacity of healthcare providers and the general population to enable planned dying [7]. In turn, how life, death, and dying is regarded has been greatly altered. Indeed, COVID-19 has reconstructed the social spheres relating to both the living and the dead, viciously challenging the modern grasp of immortality, and even proposing creative rituals of mourning, through online channels, for example, such as Zoom, Skype, and Facetime, and even through Facebook and Messenger social media platforms [8]. During the pandemic, grief seemed to have been incomplete, disenfranchised, and inadequately dealt with by the healthcare system [9]. Little is known as to what effect these new and evolving circumstances have had on nurses in general, and on end-of-life nursing care in particular.

The current study focuses on nurses who provide end-of-life care. Nurses are primary health professionals who work at the frontline of healthcare. They continuously provided care to seriously ill COVID-19 patients, while witnessing these patients’ suffering and dying, often without the physical accompanying of the patients’ loved ones, with only distance communications between them, if any [10, 11]. Of the many ethical dilemmas and significant organizational challenges that the pandemic posed for the healthcare system [12–14], the need to provide dying patients with healthcare, while assisting them with their confrontation with death in these unique and dire circumstances, were perhaps the most demanding of all.

Studies on how nurses cope with death in their practice during COVID-19 are few and limited in their scope. They reveal higher levels of fear of death, as well as increased avoidance/denial of death by attempting to ignore the topic – as a means for decreasing related stress during the pandemic [15]. In one study, more than one-third of the participating nurses stated that the pandemic had greatly changed their way of caring for dying patients. Moreover, receiving training in palliative care during the pandemic resulted in more positive attitudes towards end-of-life care among nurses [16]. In another study, while nurses showed high levels of empathy and willingness to provide holistic care, they suffered from high levels of psychological and moral distress, and acknowledged a shift towards a less patient-centered model of care [17]. Other studies referred to various gestures and personal acts of care and compassion, such as greeting patients from outside their room, making them laugh or smile, or making sure they look nice, to minimize family distress during videocalls [18].

In Israel, as with many other Western countries, the COVID-19 pandemic led to significantly increased disease and mortality rates, especially among older adults [19, 20], and before widespread and efficient vaccination took place [21, 22]. Witnessing the death of patients in the COVID-19 wards was reported to be associated with a four times greater risk of exhibiting post-traumatic stress symptoms compared to nurses from non-COVID-19 wards [23], combined with significant ethical dilemmas [12, 24].

Following this changing reality in healthcare and end-of-life, understanding how nurses experience, perceive, and interpret end-of-life care provision during the pandemic could shed light on a range of important aspects of such care, while reflecting on ethical issues, care responsibilities, and values of nurses in these circumstances. As such, this study strives to expose unique elements of the pandemic in the reshaping of nurses’ perceptions and practices of end-of-life care. Hence, the study addresses the following research question: How did the COVID-19 pandemic change or reshape nurses’ perspectives and experiences regarding end-of-life care? It aims at understanding the lived experiences of nurses who provided care during the pandemic, and the extent to which providing care under such circumstances changed or reshaped their views and perspectives about end-of-life care. More generally, the study aims at broadening the knowledge and understanding of nurses’ views and attitudes towards death, dying, and provision of end-of-life care, by addressing a range of key elements that were highlighted during the pandemic.

## Methods

### Research design

Based on the Standards for Reporting Qualitative Research (SEQR) [25], the design of this study employs the phenomenological-interpretive approach for exploring, in great detail, the participants’ lived experience and how they make sense of it [26]; the design focuses on the participants’ claims, explanatory logic, biographical contexts, and cultural frameworks of knowledge. At the core of this approach is the attempt to understand what the participants have conveyed, following their experiences; this approach, therefore, is most suitable for this research, that examines the topic of death and dying during extreme conditions (i.e., the height of the COVID-19 pandemic), and encounters meaningful questions and situations. As such, this work was informed by an ontological standpoint which focuses on *being*, namely on how the participants perceive and make sense of their experiences in the researched situation, i.e. providing end-of-life care during COVID-19. It pre-supposed that reality is relative to individual perceptions, and it is shaped by their contexts. Under this presupposition, there are multiple versions of reality because peoples’ experiences are shaped by various contexts. The research is also informed by an epistemological position, occupied by the need to *understand* human experience (end-of-life care provision) through interpretation and co-construction of meaning between participants and the researcher also observing the way that such an experience expresses itself through people’s interpretations before it is subject to concepts and norms.

#### Setting and recruitment

The research focused on the northern area of Israel not just due to convenience (as this is the district where most of the researchers work or reside), yet also since it is has the second largest population in Israel (more than 1.5 million residents) and a relatively high rate of nurses per capita compared to other areas in the country [27]. The participants were recruited using purposive and snowball sampling methods and based on their ability to elucidate the research phenomenon. Participants recruitment was based on the notion that recruiting participants who best match the research aims improves the rigor of the study and the trustworthiness of the obtained data [28]. Prior to each interview, each participant was given a printed information sheet regarding the research process, objectives, expected benefits, and possible risks to the participants. Following this information, each participant could then decide whether to decline participation in the study or sign an informed consent form and take part in an interview.

#### Data collection

In-depth semi-structured interviews were conducted with 34 nurses from eight hospitals in the north of Israel, who had provided end-of-life care during the pandemic. The interviews with the participating nurses were conducted by 4th -year nursing students from two research seminar courses that were taught and supervised by the author of this paper between October 2020-May 2022, after they had been adequately trained in the phenomenology methodology. These undergraduate students were also conducting their practical experience in hospitals in the north of Israel. All interviews were conducted in Hebrew between March 2021–February 2022, lasted 60–90 min, and were recorded and transcribed with the interviewees’ permission. Most interviews were conducted via Zoom, due to the social distancing limitations that were imposed at the time because of the pandemic. Following the phenomenological-interpretive approach, we employed interview techniques which were flexible enough to allow the participants to discuss or refer to unanticipated topics or themes. As such, the students were guided not to verify or negate with participants specific preliminary so-called truths or understandings that they had prior to the interviews while conducting the research. Moreover, the interviewers were also asked to keep an interview diary, in which they could write their reflections, pre-dispositions, and expectations, prior to and following the interviews.

To ensure consistency between interviews, a six-part interview guide was prepared by the researcher, based on the literature (See Supplementary material I). Part I included biographic questions about the participant, such as age, education, family, and work experience. Part II referred to the participants’ general perceptions and views regarding end-of-life, with questions such as, “What is end-of-life for you?” and “What do you think characterizes this phase?” Part III presented questions relating to end-of-life care. Participants were asked to share their experiences of caring for an end-of-life patient, through a series of questions such as “Please tell me about the patient’s medical background,” “What decisions did you have to make regarding this patient?” and “Which ethical dilemmas accompanied these decisions?”

The participants were also asked more general questions, such as “How did end-of-life care during COVID-19 differ from end-of-life care prior to the pandemic?” and “What thoughts do you have about the care that was provided to end-of-life patients during COVID-19?” Part IV focused on the participants’ training in end-of-life care, with questions such as, “Can you tell me about the training that you received for caring for end-of-life patients?” and “Do you feel that you would want to receive more training in end-of-life care?” Part V presented questions concerning the nurses’ external circles, such as colleagues, management, and family, with questions such as, “Who do you consult when you encounter an ethical dilemma regarding end-of-life care?” and “How do you address concerns relating to values in your personal life?” Finally, Part VI of the interview guide asked participants to talk of any issues that had not been raised by the researcher during the interview, or ask any questions that they would like to ask in light of the interview.

### Data analysis

The phenomenological-interpretive approach allowed us to search and explore narratives in two different manners: those that stem from the themes *shared* by participants and those which reflect the individual participants own accounts; we were then able to combine hermeneutics that centered on empathy and meaning recollection and hermeneutics that focused on questioning and critical engagement with the text [26]. Under such an approach the transcripts were first openly read and re-read a couple of times to annotate interesting or significant things that participants said in the interviews. Attention has been also made to new insights, the use of language and the expression of participants’ selves throughout the in-depth interviews. Then, initial annotations were transformed to concise phrases and temporary codes, adhering to participants’ exact wordings and terminology. The transformation into themes continued through the whole transcript until major themes that were identified through earlier stages were developed, made permanent, and re-organized into a scheme of codes and central themes The various categories/sub-themes were generated through a process of continuous, fluid, and recursive comparisons between and within incidents and concepts, and of theoretical coding for establishing meaning, interpretation, and understanding of the researched phenomenon. Finally, the themes were re-organized under an analytical or theoretical order, as the researcher tried to make sense of the connections between the themes, sub-themes and codes and were graphically depicted in a coding tree [29].

To increase trustworthiness [30], all interviews were analyzed discretely by the researcher and one or two students. The interviews were accompanied by a reflective diary, to secure the method of bracketing by separating between these experiences and the more general picture that is explored throughout the research [31]. Such a method does not assume that the researcher acts as having no pre-conceived ideas, free of any conceptions, aiming to reveal the “truth” in the data, but reflects reflexive engagement in the research process, while acknowledging that the interpretive task embraces considerations of multiple perspectives – including those of the researcher [32]. We facilitated a rich dialog among coders to agree on [33]. The themes, sub-themes, and codes that are presented in this article Finally, the citations presented in this paper appear in English, following their double translations, conducted by the researcher and a professional English language editor who is a native English speaker.

### Ethical considerations

The participants were informed that the findings of the study could serve for future publications. The study was approved by the Ethics Committee at the author’s affiliated academic institution (Approval# 018/20 dated 25 October 2020). Throughout this article, initials are used instead of names to present the participants’ citations, to protect their privacy.

## Results

The average (range) age of the 34 participants (20 females) in this study was 38.17 (23–52) years. At the time of the interviews, 19 of the participants were married, 13 were single and two were divorced. Their average (range) number of children was 1.5. (0–5) children. Seventeen participants were Jewish, 16 were Arabs, and one was Druze. Prior to the pandemic, the participants had worked on a range of hospital wards, including 14 in internal medicine; six in Covid-19 (including Ventilation and Recovery); four in intensive care units; two in cardiac surgery; two in pediatrics; two in rehabilitation; two in geriatrics; one in oncology; and one in palliative care. The participants had an average (range) work experience of 13.27 (1–33) years, 25 held a bachelor’s and nine held a master’s degree.

The research revealed the following five major themes: (1) a different death; (2) difficulties in caring for the body after death; (3) the need for family at end-of-life; (4) weaker enforcement of advance care directives; and (5) prolonging the dying process. The following sections will describe and exemplify these themes.

### Theme 1: a different death

The participants spoke of how dying during the pandemic greatly differed from what they had previously known. As one nurse, 27 year-old working in the ICU, explained, this was a “different death, a sad death. An unexplainable death.” The death could occur very quickly, without the presence of any family members – making it a lonely death as well. In addition, with the first two waves of COVID-19 in Israel, death was an intensive experience for nurses. As one participant, 35 year-old working in Crown (Ventilation) Department said, “over just one weekend, we had five deaths… one of the nurses [that I work with] saw two [simultaneous] deaths. The death of her patient, and the death of the person lying beside him. She didn’t show up for the next shift. She just fell apart.”

Death during the pandemic was described by the participants as “a difficult death, entailing significant suffering and pain until the very end,” and a “thirst for air”. As expressed by M., a 29-year-old nurse working in the Crown Department in a medium-sized hospital.:

A patient who up until a week ago was driving and walking, fully independent. Then just like that, he’s gone. It’s hard to believe. With COVID, they die in pain, while suffering. The hardest thing in COVID is that they die with so much suffering… They choke to death in front of your very eyes.

### Theme 2: ‘difficulties in caring for the body after death

Due to fear of COVID-19 contagion, corpses were wrapped in black plastic bags that resembled “garbage bags.” Doing so was perceived by the participants as dehumanizing and evoked anxieties: “It takes you back to the era of bombings and terror attacks [in Israel]. Those black bags, that you only saw in the newspaper… It was “awful.” (P’, 47-year-old, working in Internal Medicine Department).

Having to physically wrap the corpse was a new and disturbing experience for many nurses, that also evoked ethical concerns. Wrapping the body in a plastic bag like garbage not only objectified it, but also made in inaccessible to the living and more difficult to say goodbye. Nurses were highly deterred from carrying out such an act. As explained by T., a 27-year-old nurse working in the ICU:

When the patient dies, especially on these wards, the body is wrapped in a plastic bag, and they are buried in these bags. Everyone is afraid of COVID. The family comes to say goodbye to the deceased on the ward, before they are wrapped up [in these bags], and then they can’t see them anymore. The body is even buried in the sealed bag. That’s how it was in the second and third waves… It is very unpleasant to have to wrap a corpse. Sometimes, two people did this. Other times, four or five people, depending on the patient’s weight… When they first asked me to ‘come and help us wrap him up,’ I said, ‘What do you mean? The man is dead. Why should we wrap him up?’ The first time I did it, I thought, are we going to abuse him after he’s dead?… It’s not abuse in terms of doing something wrong, but it is showing disrespect for the body.

### Theme 3: the need for family at end-of-life

Nurses reflected how important it is for dying patients to have their families near them, and how they themselves had to fill the role of the family, when visitors were not allowed on the wards due to COVID restrictions: “To feel like there’s someone there also helps the family. That’s care in itself. Just being there, holding their hand, letting them know that they’re not alone. That’s the best care, because nothing else could help them anyway” (H’, a 57-year old, working in Geriatrics Department). The absence of family members led to the expansion of the nurses’ roles, as they found themselves filling this role as well –not only by providing patients with emotional support and encouragement in these difficult times, but also assisting with daily issues. As articulated by Z., a 49-year-old nurse from Internal Medicine:

One day I brought ice-cream for a patient and things like that, you know, all sorts of trivial things. There was one patient who wanted coffee, so when I went downstairs and brought her a cup of coffee. There was another patient whose shoes disappeared, so I bought him a new pair of shoes. We really stood in for the family throughout this period.

In addition, not all family members of deceased patients came to say goodbye to them after they died, as they had to wear protective clothing to do so, which was difficult or annoying for some. N. (aged 28, Crown and Recovery) shared how such situations instilled in her the feeling of a privilege, being beside these patients at their end-of-life:

It’s like a privilege… in his last moments as a human being, to be next to him, to cover him. It’s that moment, it’s not a simple moment, not everyone is up to it, but it is a very meaningful moment. I felt meaningful. Not his family, not his children, not his wife. But me. I’m here [beside him] during his last moments [alive].

The pandemic highlights the importance of the presence of the patient’s family in end-of-life from a range of aspects. First, with COVID-19, patients often deteriorated quickly and unexpectedly. Yet as the family was absent due to restrictions, the staff was not always able to inform the family about this worsening of symptoms in time. This led to feelings of frustration among the nurses; there was no “closure” at the end-of-life, unlike other situations where a patient is dying. In addition, some participants explained that in many cases, the family actually helps the staff: in calming down the patient, while interpreting the situation and informing the patient about the medical diagnosis in a more informal and less medical language. Yet during the pandemic, not only was the family not there to fill these roles, but the nursing staff also had to fill the additional role of liaising between the patients and their remote families in such difficult times.

Third, in many interviews, the nurses expressed how the absence of family seemed to have compromised the patients’ care and even hastened their death: “The family knows the patient better than the medical staff. They are a vital resource.” The participants even shared that in some cases, they did not understand what the patient wanted, or which treatment the patient had received in the past – for comprehensively understanding the patient’s medical anamnesis (i.e., medical history). As a result, the care provided to these patients may not have always been optimal or efficient. In addition, patients suffered from lack of communication with their loved ones, despite the nurses’ attempts to enhance communication between patients and their families through technological means – especially mobile devices such as phones and tablets. Yet despite such efforts, some patients felt abandoned by their families, due to physical restrictions that were enforced throughout the country during the pandemic. As explained by R. (aged 33, Internal Medicine):

In our department, we had this tablet that we could use for conducting video conferences between patients and their families. But the patients often refused to speak to their families this way. They felt sad, and even abandoned. This was really hard on patients who were depressed or mentally unstable. They often also became physically ill, by refusing to eat, for example.

Finally, the participants emphasized how family presence is crucial for saying goodbye and supporting patient in their last moments, as explained by I. (aged 47, Internal Medicine):

You know, we have patients who hang on until they meet their family. For example, the grandchild who returns from overseas and gets to see the patient and truly say goodbye. The patient waits for that. This is a very important thing, to truly say goodbye to the patient. But that was impossible during COVID.

I. also explained how such situations impacted the staff’s complex attitude towards the patient’s family:

I’m aware that the mental and psychological state of many patients deteriorated while they were hospitalized on the COVID-19 ward, because of their ongoing disconnect from their family. We understand the importance of family and whenever we could, we let the family into the patient’s room wearing protective clothing, even if it was just to stand there for a few moments in the patient’s final hours.

Both S. (aged 50, Children and Crown) and Y. (aged 23, Internal Medicine) also spoke about their difficulty as nurses to connect with patients without the assistance of their family members and to witness the lack of physical touch by family members, saying:

Sometimes, we’re critical of the patient’s family, because we think they’re getting in the way of our work. But in these difficult moments, they are really missed. It even affects our ability to connect with the patients. (S.)

Families are an integral part of the triad: patient – healthcare provider –family. I don’t recall any situation in the past where the family was kept away from a terminally ill patient. In many cases, their only communication took place via technology. But there’s no substitute for the touch of a hand or a hug of encouragement from a family member. It was difficult for us [nurses] as well, watching this from the side. (Y.)

### Theme 4: weaker enforcement of advance care directives

Advance care directives strive to decrease conflicts and increase autonomy at end-of-life. Yet interviews with the nurses reveal that during the pandemic, these directives were not fully enforced by the healthcare teams. There was a type of attempt to keep patients alive, with resuscitation serving as a default, despite other directives: “You want me to resuscitate a corpse? A corpse that’s been expiring for the last 24 hours. Now you want to ‘help him’ instead of making things easier for him?” (T’, a 27-year old, working in the ICU). As expressed by S. (aged 50, Children and Crown):

Sadly, there was this atmosphere in the department of ‘just keep them alive.’ And that was also a question. How much do we harm the patient vis-à-vis the principle of beneficence. What are the patient’s chances of being weaned off this artificial respiration and regaining full (or even partial) functioning? Unfortunately, there were DNRs [Do Not Resuscitate advance care directives for end-of-life], but I don’t think that they [the medical teams] sufficiently respected them. They really provided respiration for everyone, really everyone.

Another explanation for not fully enforcing advance care directives relates to the unexpected development/regression of the disease in patients with COVID-19, as explained by T. (aged, Ventilation):

The family was asked whether to resuscitate or not, even when the staff on the ward knew that there were advance care directives, and despite that, they acted according to the family’s decision. Obviously, when they see a patient in such circumstances, when they’re not prepared for it, they don’t, they don’t…even if you think of these things, you are not really ready, ready for this in real time. So in the end, the patient is resuscitated.

### Theme 5: prolonging the dying process

The nurses felt that prior to the pandemic, they provided more conservative care and administered medication to facilitate a more comfortable dying process; yet the pandemic provoked more intensive and aggressive care, and in many instances, even more than necessary. The participants explained that less palliative care was offered to patients, due to the uncertainty and fluidity of their clinical status. “The disease was new, and we didn’t know how to treat patients, so we provided care to all patients, giving the maximum aggressive care. Almost everyone was resuscitated. People received care like in the ICU [intensive care unit], with no difference between patients.” (A’, Aged 52, ICU). Yet since the staff was not sure whether the patient would recover or not, or whether they were terminally ill, they worried about subjecting all patients to aggressive care – yet such care was provided to all patients, including elderly patients with co-morbidities, as conveyed by R. (aged 33, Internal Medicine):

There were patients whose conditions were very difficult, and they had many background diseases, and they were very old. We just fought with them and gave them every possible treatment that existed in the hospital. We hooked them up to lots of tubes, gave them units of blood, almost everything. It was as if we knew that this is it, the prognosis is bad, but because they also had a disease [COVID-19] and this was at the beginning of the pandemic, we gave them everything.

Yet as time went by, and with additional waves of the pandemic, the participants admitted that there was a certain, albeit small, degree of change in the staff’s attitude towards care, although not a dramatic one. “I was working as a nurse during the third wave of COVID… The attitude had slightly changed. We still gave aggressive care but to a lesser extent. At some point we would say, ‘that’s enough, we shouldn’t do anything else.’” (H’, aged 34, Internal Medicine).

Such situations provoked moral distress among nurses. As explained by T. (aged 24, Ventilation):

This is very difficult decision making. In retrospect, it’s very easy to say, ‘there was a clear and final answer,’ but when you’re there, you don’t want to be the person who says to the family, ‘let’s leave it…’ And that’s difficult.

Part of the nurses’ distress could be attributed to their often having to provide futile treatment, even though they didn’t believe in what they were doing. As R. (aged 26, Palliative Care) explained: “It was unpleasant performing these resuscitations. Completely unnecessary. It’s not even pleasant saying this out loud. And so uncomfortable for the staff. Performing actions and procedures that you know are pointless. Hmmm.”

## Disscussion

The aims of the study were to explore nurses’ lived experiences while providing care to patients at end-of-life during COVID-19, and to examine changes in their perspectives on end-of-life care following the COVID-19 pandemic. The five emerging themes offer rich insights that shed light on the broad challenges that this contagious and widespread disease imposed on nurses, who had to provide urgent care in extreme unfamiliar circumstances [24]. As explored in other studies, nurses in Israel witnessed numerous cases of death and dying during the pandemic, while facing significant ethical and professional issues regarding end-of-life care. This was an unprecedented and devastating experience [11, 34], that as with other healthcare professionals, likely also increased the already high rates of nurses’ work-related stressors, burnout, boundary ambiguity, isolation, financial constraints, and overall frustration [35, 36] Unlike other countries, where studies indicate that these situations were managed as a collective mission and purposeful action reflected by the term “community of fate” [37], the nurses in this study spoke of more individualistic, private, and personal ways through which they processed and responded to these events. This may be due to lack of professional, organizational, and collegial support [12], and more generally, following the influence of working in a neoliberal healthcare context that places the individual at the centre [38, 39].

The study also uncovers the various additional roles that nurses filled when the patient’s family members were not able to be physically present because of COVID-19 restrictions. They provided extended emotional support for both the patient and the family; facilitated communications between them; and substituted for the family during the patient’s last moments alive. This study, along with other studies conducted during the pandemic [37], also sheds light on the key role and place of the family in the patient’s final moments. While the literature emphasizes the family’s contribution to the patient’s end-of-life and their need to say goodbye to the patient as part of the mourning process [40, 41], this study is novel in that it demonstrates that the presence of the family at the patient’s end-of-life is important for the nurses as well in two important ways. First, it facilitates patient-nurse communications, especially when these are challenged due to health-related considerations (i.e., pandemic restriction). Moreover, the presence of family members provides the physical touch and comfort required by patients in difficult times, thereby relieving nurses from additional pain and distress while carrying out their roles.

In addition, the findings of this study reflect the moral distress experienced by the nurses, who were instructed to perform resuscitation or respiration, even when such procedures were futile or contrary to the patient’s advance directives. The nurses in this study expressed how the ethos of saving lives and “doing whatever is possible” was dominant, especially during the early phases of the pandemic. While such ethos is reflected in additional studies on end-of-life care in Israel [42, 43], this study substantially highlights its detrimental effect on the nurses, as one of the main characteristics of moral distress [44–46]. Our findings indicate that end-of-life care was more aggressive than required, thereby leading to increased moral distress among the nurses. Providing such care weighed heavily on them, as seen in a previous study, where nurses reported distress, anxiety, and sleep disorders [47]. This adds to previous findings, whereby nurses perceived end-of-life care during COVID-19 as lacking in spirituality, empathy, and compassion [48]. While consensus is lacking regarding the inadequate end-of-life care as a source of moral distress during the pandemic, studies emphasize that nurses struggled to have their voices heard in this regard [49]. Yet this was not seen in the current study.

The aggressive end-of-life care resulted in resuscitation as a default, thereby also invalidating patients’ advance directives or prior requests asking for the opposite, in similar circumstances. This finding, which may be influenced by cultural attitudes toward the sanctity of life and its dominance over personal autonomy [50], is contradictory to studies conducted outside Israel, whereby during the pandemic, advance care planning and palliative care consultations were associated with care de-escalation [51] and less morbidity [52], and thereby more frequently enforced. Yet similar to other studies [53], the findings of the current study indicate that when such directive/specific requests were absent, they could not be formed due to the rapid deterioration of the disease and the difficulty to plan for future care. Other reasons for not drafting advance directives during the pandemic were not referred to in the interviews, such as social distancing restrictions and remote communications with family members, extensive workloads, staff sickness and shortages, the need to use personal protective equipment adding time and workload for each face-to-face interaction with patients, lack of coordination between healthcare professionals, and national contexts of fear and anxiety [53–55].

Nurses’ ethical concerns were also triggered by special requirements to handle the corpse, due to the pandemic. Such demands were perceived as abusive and disrespectful towards the dead. Although such requirements were also addressed by nurses in other countries [11, 37], at times even reflecting normalization processes by which nurses self-justify their need to provide a committed response to the pandemic demands [56], they did not provoke similar emotional and ethical responses. This unique finding could be explained by the fact that social and cultural differences impact nurses’ perceptions on end-of-life care in general, and by the role that death and the deceased play in certain religions such as Judaism [57], as well as the cultural values of healthcare providers in Israel [58] more specifically.

Finally, while death is a social phenomenon, and the perception of death and dying is culturally influenced [59], it also comprises basic universal elements. The perspectives explored in this research attest to the importance of nurses’ self-awareness and consideration of their emotional states and attitudes in difficult and challenging situations, and offer a bridge for their understandings, especially in relation to the unique circumstances of the pandemic. They also demonstrate the importance of relationship-centered care in end-of-life and palliative care, which was significantly disrupted during the pandemic [60].

## Implications on policy and practice

The literature emphasizes the need to develop institutional, organizational, and peer support as a means for responding to difficult events in the healthcare system, such as the COVID-19 pandemic [61, 62]. Yet the findings of this study emphasize that nurses may respond to such events in an individualistic, private, or personal manner. It is therefore important to help nurses strengthen their resilience and capabilities regarding self-help and self-monitoring – in such s way as to be able to reflect on their challenges, successes, and failures to meet these goals and to learn from such reflections. An additional implication of this research relates to the contribution of the patient’s family to the wellbeing of the nurses themselves, especially when providing end-of-life care. Nurses should be able to articulate and better understand the ways in which family members can be involved in such a care, to maximize their own satisfaction and sense of worth. Nurse educators, policy makers, and healthcare managers should incorporate these findings into their programs, to ensure the more comprehensive education of nurses regarding end-of-life care that better reflects their perceptions and meanings.

## Limitations

The study may have some limitations. First, the interviews and data analysis were conducted by a relatively large number of people who have different interview styles and approaches to the research at stake. While, on a first look, this may be considered a limitation, it is important to emphasize that all the students who interviewed for this study were trained and qualified by the same author, and that the author was personally and fully involved in the data analysis. More generally, triangulation of researchers not only shows the complexity of the researched social reality, but also secures an important element of trustworthiness. allowing for a rich reflection and interpretation on data, thereby increasing confidence in the outcomes [63]. Second, the study used purposive and snowball sampling to recruit participants. While such sampling methods may seem selective and biased, the relatively large size of the study sample minimizes these risks by providing a richer and fuller picture of the researched phenomenon as it is understood and conveyed by more members in the research population. More generally, these methods are most popular in qualitative research due to their ability to access participants, especially exploring difficult or sensitive issues like death and dying, and are supported by the idea of social networking which suits well with the key premises of qualitative research [64]. Third, the dialog between the researcher and his students required for the analysis process may suffer from some limitations [65]. Specifically, high levels of agreement between researchers regarding the codes and data analysis could have stemmed from interpersonal dynamics and the fact that the first author is more knowledgeable about the research project that the students, and as such may have had more established expectations of the achieved input [33]. However, during the negotiated agreement method, in some cases, the students’ thematic coding was adopted. Moreover, these potential challenges were discussed in class, prior to conducting the data analysis, so that the students were familiar with them beforehand and could better manage them, if and when needed. CONCLUSIONS.

Nurses who provided patients with end-of-life care during COVID-19 experienced significant emotional, ethical, and professional challenges. The unique conditions posed by the pandemic reshaped nurses’ perceptions of death and dying as a lonely and even disrespectful process. Often at the cost of the nurses’ own wellbeing, such conditions enhanced the complexity of their relationship with the patient’s family, liaising between family and patient, and even standing in for the family during the patient’s final moments. Care provided to such patients involved extending life-saving treatment, contrary to the patient’s explicit wishes or to the nurses’ perceptions of its effectiveness. Combined with having to wrap the bodies in bags, the nurses in the study experienced moral distress and strong moral sentiments linked to their work and experiences during these tough circumstances and associated with practices of wrapping the patients’ bodies, providing futile end-of-life care to patients, not enforcing advance directives, and so forth. All in all, they addressed a range of important issues, thereby raising their self-awareness and repositioning their attitudes towards the nursing profession and themselves.

### Electronic supplementary material

Below is the link to the electronic supplementary material.

Supplementary Material 1

## Data Availability

The datasets used and/or analysed during the current study are available from the corresponding author on reasonable request.
