# Supplementary material for: “They choke to death in front of your very eyes”: nurses’ lived experiences and perspectives on end-of-life care during COVID-19
Source: BMC Palliat Care. 2024 Feb 8;23:35. doi: 10.1186/s12904-024-01352-3 (PMC10854065; doi:10.1186/s12904-024-01352-3)
Supplement: Supplementary file 1 — Supplementary Material 1 [file 12904_2024_1352_MOESM1_ESM.docx]

**ANNEX A – Interview Guide**

**Part 1- Biographical questions**

Please tell me about the following biographical characteristics of yours: age, family and educational background, field of expertise, clinical background, years of experience in the hospital, additional responsibilities, etc.

**Part 2 – General perceptions and views regarding end-of-life**

What is end-of-life for you?

In your views, what do you think characterizes this stage?

To what extent do you think that these characteristics have changed because of COVID-19? Please explain how.

Can you please share with me your thoughts pertaining to this stage?

**Part 3 – End-of-life care**

Please share with me your experience in caring for patients who are at end-of-life, especially during COVID-19. You may refer to one or more situations:

What was the patient background?

What was he/she diagnosed with?

What was your professional responsibility?

What decisions did you have to make regarding this patient?

Were you asked to consult with other people and if so, with whom?

What was the role of the patient's family in the decision making process?

Did the decision making process involve ethical dilemmas? If so, please describe them in detail.

Which values were involved in the decision making?

How did you make a final decision?

Why did you decide this way?

Are you satisfied with your decision? Why?

To what extent you think that your experience in end-of-life care during COVID-19 is different than caring for other patients at this time? If so, please explain how is it different.

Can you please share with me a situation in which you had to say goodbye to a patient during COVID-19, if there was any?

What thoughts do you have about the care that was provided to end-of-life patients during COVID-19?

What do you think about life-saving treatment offered today to patients with COVID-19?

How does the management of end-of-life care affect you personally?

**Part 4 – Training in end-of-life care**

Did you receive any training on end-of-life care? If so, where? And what was its extent?

Did you receive any training on end-of-life care during COVID-19?

Do you feel that you would want to receive more training in end-of-life care?

What would you like to receive training on?

How do you think training on end-of-life care/ethical dilemmas at end-of-life should take place?

**Part 5 - External circles**

Who do you consult when you encounter an ethical dilemma regarding end-of-life care?

To what extent is your supervisor involved in ethical questions related to your professional decision?

How do you address concerns relating to values in your personal life?

Would you have wanted to share your thoughts/dilemmas with other people? If so, with whom?

**Part 6 – Summary and Epilogue**

Are there any issues that had not been raised by me during the interview?

Did you have any new thoughts during the interview that you would like to share them with me?

Are there any questions that you would like to ask in light of the interview?

With whom would you have recommended me to speak on my research subject?
